# Testing Efficacy of a Conserved Polypeptide from the Bm86 Protein against *Rhipicephalus microplus* in the Mexican Tropics

**DOI:** 10.3390/vaccines11071267

**Published:** 2023-07-21

**Authors:** Raymundo Coate, Miguel Ángel Alonso-Díaz, Moisés Martínez-Velázquez, Edgar Castro-Saines, Rubén Hernández-Ortiz, Rodolfo Lagunes-Quintanilla

**Affiliations:** 1 Facultad de Medicina Veterinaria y Zootecnia, Universidad Nacional Autónoma de México, Avenida Universidad 3000, Ciudad de México 04510, Mexico; coate_rc@comunidad.unam.mx; 2 Centro de Enseñanza, Investigación y Extensión en Ganadería Tropical, Facultad de Medicina Veterinaria y Zootecnia, Universidad Nacional Autónoma de México, Km. 5.5 Carretera Federal Tlapacoyan-Martínez de La Torre, Martínez de La Torre 93600, Mexico; alonsodm@unam.mx; 3 Centro de Investigación y Asistencia en Tecnología y Diseño del Estado de Jalisco, A.C, Avenida Normalistas 800, Col. Colinas de la Normal, Guadalajara 44270, Mexico; mmartinez@ciatej.mx; 4 Centro Nacional de Investigación Disciplinaria en Salud Animal e Inocuidad—INIFAP, Carretera Federal Cuernavaca—Cuautla 8534, Col. Progreso, Jiutepec 62550, Mexico; castro.edgar@inifap.gob.mx (E.C.-S.); hernandez.ruben@inifap.gob.mx (R.H.-O.)

**Keywords:** anti-tick vaccine, Bm86, polypeptide, *Rhipicephalus microplus*

## Abstract

*Rhipicephalus microplus* economically impacts cattle production in tropical and subtropical countries. Application of acaricides constitutes the major control method; however, inadequate use has increased resistant tick populations, resulting in environmental and cattle product contamination. Anti-tick vaccines based on the Bm86 antigen are an environmentally friendly, safe, and economically sustainable alternative for controlling *R. microplus* infestations. Nevertheless, variable efficacy has been experienced against different geographic tick strains. Herein, we evaluated the efficacy of a conserved polypeptide Bm86 derived from a Mexican *R. microplus* strain previously characterized. Twelve cows were assigned to three experimental groups and immunized with three doses of the polypeptide Bm86 (pBm86), adjuvant/saline alone, and Bm86 antigen (control +), respectively. Specific IgG antibody levels were measured by ELISA and confirmed by Western blot. In addition, the reproductive performance of naturally infested *R. microplus* was also determined. The more affected parameter was the adult female tick number, with a reduction of 44% by the pBm86 compared to the controls (*p* < 0.05), showing a vaccine efficacy of 58%. Anti-pBm86 IgG antibodies were immunogenic and capable of recognizing the native Bm86 protein in the eggs, larvae, and guts of *R. microplus*. The negative correlation between antibody levels and the reduction of naturally tick-infested cattle suggested that the effect of the polypeptide Bm86 was attributed to the antibody response in immunized cattle. In conclusion, the polypeptide Bm86 showed a specific immune response in cattle and conferred protection against *R. microplus* in a Mexican tropical region. These findings support further experiments with this antigen to demonstrate its effectiveness as a regional vaccine.

## 1. Introduction

*Rhipicephalus microplus* is the principal vector in cattle production due to the direct skin lesions caused by the hematophagous action and indirect effect as a biological or mechanical vector of multiple diseases, such as babesiosis and anaplasmosis [1,2]. *R. microplus* is distributed in the tropical and subtropical countries of Africa, Australia, and Latin America (32° N, 32° S), which is the major challenge to the cattle industry, affecting milk and meat production [3]. In this context, Mexico is located between the parallels 32° N y 14° S, where the climate is predominantly humid-tropical and presents the appropriate conditions for the recurrent appearance of tick infestations [4,5]. As a result, at least 4 million cattle heads in the Mexican tropics [6] are exposed to damage, affecting livestock health and the economy of ranchers [2,7]. Furthermore, these infestations are not limited to these regions; national economic losses are estimated at around USD 573,608,076 [5].

For decades, *R. microplus* has been controlled by acaricides. However, inadequate and excessive use has occasioned the appearance of multi-resistant strains. The Mexican market distributes organophosphates, pyrethroids, amidines, phenylpyrazolones, IGRs, and macrocyclic lactones. Nevertheless, they have been surpassed by the genetic plasticity shown by *R. microplus* [8,9]. Hence, alternative approaches have been proposed, including pasture rotation, selection of resistant cattle (*Bos indicus*), and biological control using tick-predatory organisms, such as fungi, nematodes, bacteria, and arthropods [10,11,12,13]; most of these practices are desirable to reduce chemical residues in cattle products and the environment [14]. On the other hand, immunological control is one of the most important approaches developed since the 1990s and uses the Bm86 antigen, a glycoprotein located at the epithelial cell membrane of the *R. microplus* tick gut [15,16,17]. Commercial tick vaccines have been distributed as TickGARD^TM^ (Australia), GAVAC^TM^ (Latin America), and, more recently, Bovimune Ixovac^TM^ (Mexico). These vaccines use *Escherichia coli* or *Pichia pastoris* as recombinant expression platforms that are friendly to the environment and do not contaminate milk and meat products [18,19].

On broad literature analysis, numerous tests have been performed with Bm86, and variable efficacy between 45% and 100% controlled infestations with *R. microplus*, *R. annulatus,* and *R. decoloratus* strains have been experienced [3,18,19,20,21] due to sequence variations (>2.8%) in the target protein among different tick strains in America [22]. These patterns also have been observed in the Bm86 protein in Mexican *R. microplus* strains [23]. Nevertheless, field trials with Bm86-derived vaccines have been established in Latin America and Australia as an integrated tick management program; the program controlled *R. microplus* infestations through reduced tick feeding, decreased reproductive capacity and egg lying, and the reduction of larvae in the field in subsequent generations [15,24].

Following the premise that the Bm86 sequence is variable, developing immunogens based on the complete protein of each local strain appears to be a viable and logical strategy. However, it is likely to be a slow undertaking with limited scope in terms of resources and future expectations. In contrast, vaccines developed from conserved regions across tick strains and potential immunogenic activity appear desirable to avoid the variability observed in the Bm86 protein [23,25,26].

Currently, different approaches are being considered for identifying and characterizing candidate tick antigens; some of these proteins are conserved across the tick species, involved in blood digestion, pathogen transmissions, and tick-host-pathogen interface, and should be accessible to the host immune system [14,27]. Therefore, vaccines based on this strategy have been designed to focus on hydrophobicity, antigenicity, cellular localization, motifs to predict regions with immunogenic potential, and epitopes recognized by B and T cells that could elicit specific humoral and cellular immune responses characterized by IgG antibodies and the complement proteins [28,29,30].

Studies of the Bm86 protein revealed that it contains antigenic epitopes, which presented within 20 different South American tick strains [31], and one of which was tested as a synthetic peptide and showed significant IgG levels and an efficacy of 81.05% in cattle against *R. microplus* [32]. Moreover, in silico analysis predicted conserved epitopes in the Bm86 protein from different geographical regions using a Brazilian Bm86 sequence of the *R. microplus* strain as a reference [26]. Additionally, some experimental vaccines based on the orthologous Bm86 have successfully controlled infestation with *R. microplus*, *R. australis*, *R. annulatus,* and *R. decoloratus* [33,34,35].

The approach used in this study is based on previous bioinformatics and molecular analysis of a polypeptide derived from the Bm86 protein, performed in a Mexican *R. microplus* strain. This immunogenic region consists of 178 amino acids and a 98–100% identity/similarity concerning strains of *R. microplus* ticks from the Americas [36]. Our findings may become valuable for developing a regional vaccine against *R. microplus*. Thus, this work aims to evaluate the efficacy of a conserved polypeptide derived from the Bm86 in cattle to improve and propose a new formulation against *R. microplus* infestations in the Mexican tropics.

## 2. Materials and Methods

### 2.1. Ticks

The susceptible “Media Joya” strain of *R. microplus* was obtained from laboratory colonies kept at the Tick Laboratory of the National Center of Disciplinary Research in Animal Health and Safety from the National Institute for Forestry, Agricultural and Livestock Research (CENID-SAI, INIFAP) in Jiutepec, Morelos, Mexico. This tick strain was collected originally from cattle infested in Tapalpa, Jalisco, Mexico.

### 2.2. Cloning, Expression, and Purification of Recombinant Polypeptide Bm86

The cloning of the experimental Bm86 fragment in *Escherichia coli* was done as described previously [36]. In short: the encoding Bm86 fragment was cloned using the expression vector pET101/D-TOPO^®^ (Invitrogen, Waltham, MA, USA). The recombinant constructs were then transformed into One Shot^®^ Top 10 *E. coli* cells (Invitrogen, Waltham, MA, USA) according to the standardized instructions provided by the fabricant.

For expression of the recombinant polypeptide Bm86, plasmids were transformed into *E. coli* BL21 Star (DE3), propagated in Luria-Bertani (LB) broth with ampicillin (50 μg/mL) and cultured at 37 °C. The recombinant synthesis was induced with 1 mM of isopropyl-beta-D-thiogalactopyranoside (IPTG) for 5 h. The cells were disrupted by sonication six times at 40 Hz on ice, centrifuged at 14,000× *g* for 10 min at 4 °C, and stored at −20 °C. The recombinant expression was confirmed by 15% SDS-PAGE gels stained with Coomassie Brilliant Blue. Western blot analysis was performed throughgels that were transferred to polyvinylidene difluoride (PVDF) membranes and blocked in TBS containing 0.05% of Tween-20% (TBS-T) and 5% skim milk for 1 h at room temperature and then overnight at 4 °C. An anti-His monoclonal antibody (Invitrogen) alkaline phosphatase conjugate was used 1:2000 to detect the recombinant His6x-fusion protein. After three washes with TBS-T, the color was developed using a BCIP/NBT alkaline phosphatase substrate (Millipore, Billerica, MA, USA). The recombinant proteins were purified under denatured conditions with a NI-NTA spin kit (Qiagen, Hilden, Germany) by Ni affinity chromatography according to the method recommended by the manufacturer.

### 2.3. Location and Cattle

The field trial was carried out in the Centre for Teaching, Research and Extension in Tropical Livestock (CEIEGT) from the National Autonomous University of Mexico (UNAM) (20°02′ N, 97°06′ W) in Martinez de la Torre, Veracruz, Mexico. The study area has a humid tropical climate with an average daily temperature of 23.4 °C, relative humidity of 85%, and annual rainfall of 1743 mm [37]. Twelve crossbred cows (*Bos taurus* × *Bos indicus*), aged 6–7 years with an average weight of 600 kg and prior exposure to *R. microplus* infestation, were used for the experiment. The cattle were apparently healthy and raised in an extensive grazing system on native grasses and water ad libitum. Animals had not been treated with acaricides or been pregnant or nursing calves in the preceding month. All the cows in this study were handled according to the Institutional Animal Care and Use Committee (SICUAE) of the Faculty of Veterinary Medicine and Zootechnics (FMVZ) from the UNAM and supervised by a veterinarian.

### 2.4. Immunization Experiment

Cattle were randomly divided into three experimental groups of four cows each. Group 1 was immunized with the polypeptide Bm86 emulsified with Montanide ISA 50 V oil adjuvant (Seppic, France). The cows of group 2 were injected with the recombinant Bm86 antigen as a positive control. Group 3 received an emulsion composed of 1 mL of phosphate-buffered saline (PBS) solution plus 1 mL of Montanide ISA 50 V per dose and were considered control. Cows in the immunized groups received three doses containing 100 μg/2 mL at days 0, 30, and 49 via subcutaneous injection. All animals were monitored for local reactions or clinical signs post-immunization.

### 2.5. Cattle Blood Collection

Throughout 63 days, blood samples were obtained from all 12 animals at weekly intervals. Samples were collected from the caudal vein using sterile tubes before each immunization and post-immunization period. The blood samples were centrifuged at 4000 rpm for 15 min at 25 °C, and serum was separated and stored at –20 °C until further analysis by indirect ELISA and Western blot.

### 2.6. ELISA Antibody Serology

Purified recombinant proteins were obtained according to the previous procedure. ELISA plates were coated overnight at 4 °C with 1 μg/well of the antigens. Subsequently, the wells were washed three times using PBS containing 0.05% of Tween-20% (PBS-T), blocked with 5% skim milk in PBS-T, and incubated for 1 h at room temperature. Following three additional washes with PBS-T, the plates were incubated with sera samples diluted 1:100 in PBS-T for 1 h at room temperature and then incubated with 1:2000 anti-bovine IgG-AP conjugate (Sigma, St. Louis, MO, USA) for 1 h at room temperature. The color reaction was developed using 3,3′,5,5′-tetramethylbenzidine (Sigma), and the optical density (OD) was measured at 405 nm. Finally, antibody levels were considered positive when the OD_405_ nm value was at least twice as high as the pre-immune serum and were compared using a one-way ANOVA test (*p <* 0.05).

### 2.7. Detection of Native Bm86 Protein in R. microplus Tissues by Western Blotting

A total of five ingurgitated *R. microplus* female ticks were collected and dissected to separate the guts using fine forceps, washed twice in PBS, and stored at –70 °C. Additionally, 100 mg of larvae and egg mass were pulverized using a frozen mortar and placed in an Eppendorf tube with 1 mL of PBS. The extracts were prepared following the method described by Popara [38]. Protein concentrations were determined by a Bradford protein assay using bovine serum albumin as a reference standard. Afterward, protein extracts were separated using 15% SDS-PAGE gels and transferred to PVDF membranes. The PVDF sheets were blocked in PBS containing 0.05% of Tween-20% (PBS-T) and 5% skim milk for 1 h at 4 °C with gentle rocking. After three washes with PBS-T, the membranes were cut into strips (~0.4 mm) and incubated with serum from one representative bovine per experimental group, collected on day 35. The serum was diluted 1:300 in PBS-T and incubated for 2 h at 4 °C. Then, strips were washed three times and incubated with anti-bovine IgG-AP conjugate (Sigma, St. Louis, MO, USA) diluted 1:2000 in PBS-T for 2 h at room temperature. The positive signal was developed using a BCIP/NBT alkaline phosphatase substrate (Millipore, Billerica, MA, USA).

### 2.8. Data Collection and Evaluation

**Figure 1:**
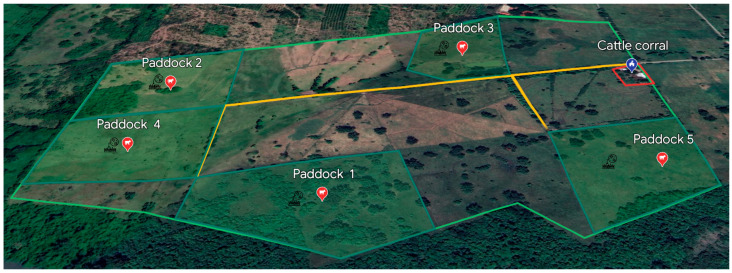
Spatial distribution of cattle in paddocks at CEIEGT-UNAM (Rancho “El Clarín”), Veracruz, Mexico.

The animals were kept in five paddocks fenced with barbed wire and naturally infested with *R. microplus*. The paddock rotation was considered; the cows roamed free in paddock 1, followed by 2,3,4, and 5, respectively (Figure 1). The cattle grazed in the paddocks for between 7 and 10 days.

To assess the vaccination effects on tick biology, adult female ticks (4.5–8 mm) were collected weekly on one side of the animal and multiplying by two to obtain the total number of adult ticks per animal [39]. All the ticks collected were counted and individually weighted and placed in tick chambers maintained at 27 °C and 80% relative humidity to allow oviposition and larvae hatching. The vaccine efficacy was measured based on reduced number of adult female ticks, tick weight, oviposition, and egg fertility. The percent reduction was calculated with respect to the control group using the standardized formula [34,40].

### 2.9. Statistical Analysis

The statistic comparison of tick number, tick weight, oviposition, and egg fertility for each group was analyzed using a one-way ANOVA test. A least significant difference (LSD) test was applied to verify the differences between vaccinated and control cattle, and data with *p* < 0.05 were considered statistically significant. The results are presented as average ± standard deviation (SD). Additionally, a correlation analysis was performed using Spearman’s test to compare the number of ticks collected after feeding with the antibody levels over time in each cow. These statistical procedures were carried out using StatGraphics^®^ software (version 19.1.3).

## 3. Results

### 3.1. Characterization and Production of Recombinant Polypeptide Bm86

**Figure 2:**
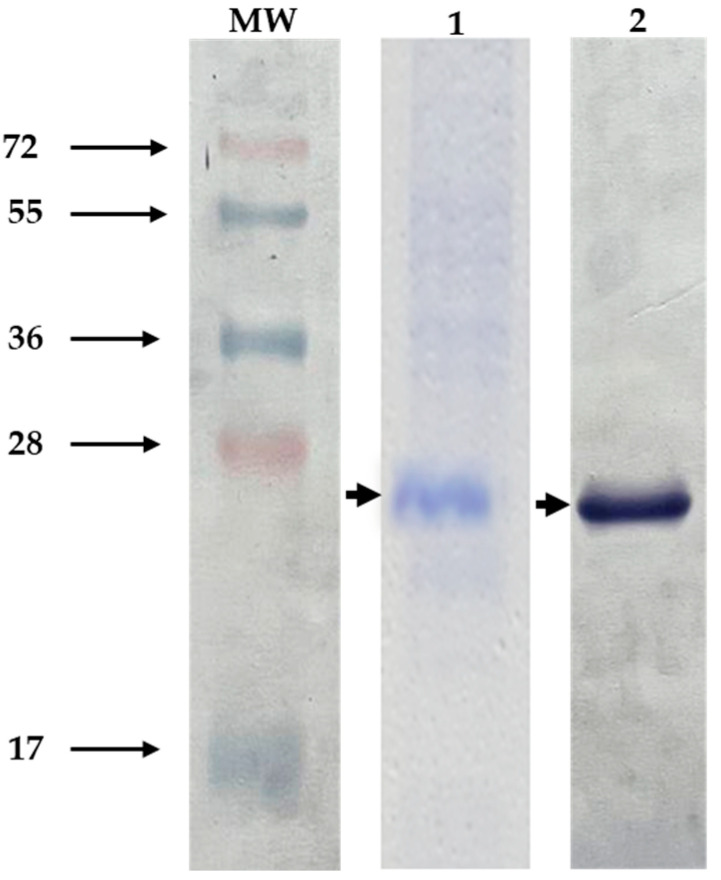
Recombinant polypeptide Bm86 expression. Protein samples containing the recombinant pBm86 were analyzed by 15% SDS-PAGE gels stained with Coomassie Blue and Western blotting. (1) induced *E. coli* cultures with IPTG expressing the recombinant polypeptide Bm86. (2) Western blot probed with anti-His monoclonal antibody (1:2000). Arrows indicate the size of the recombinant Bm86. MW, molecular weight.

The sequence for the Bm86 fragment encodes 178 amino acids and was derived from the *R. microplus* “Media Joya” tick strain. The immunogenic region selected was expressed in *E. coli* as an inclusion body, solubilized using 8 M urea, and purified by His-affinity chromatography. After induction, expression levels of the polypeptide Bm86 reached approximately 10% of total cellular proteins. The molecular size was approximately 26 kDa, based on visualization on SDS-PAGE gels and Western blot using an anti-His monoclonal antibody recognized the His6x-fusion protein on the C-terminal of the pBm86 (Figure 2). The purified recombinant polypeptide Bm86 was successfully obtained, and the sample purity was analyzed and estimated to be 90% pure.

### 3.2. Effect of Immunization on Biological Parameters of R. microplus Ticks

**Figure 3:**
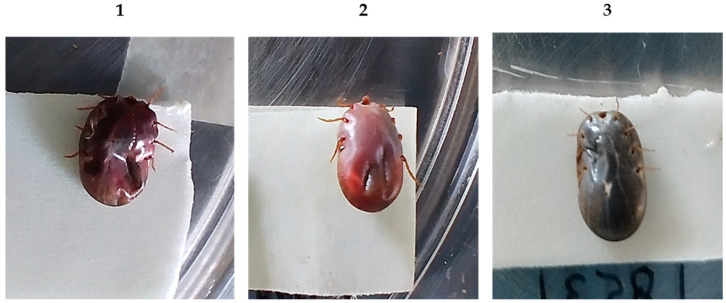
Ticks were collected from experimental groups on day 56. The damaged tick engorged on (**1**) polypeptide Bm86 and (**2**) Bm86 showed a characteristic dark-red coloration. (**3**) Ticks collected from controls.

Table 1 shows the effect on the biological parameters of engorged adult female *R. microplus* ticks collected from the cattle immunized. The immunization of cattle with the polypeptide Bm86 and Bm86 antigen reduced the number of ticks engorged on the animals by 44% and 48%, respectively, compared to the control group (*p* < 0.05). Additionally, several ticks collected from both experimental groups were visibly damaged and showed effects such as physical damage, dry appearance, a dark-red coloration, and, in some cases, death was observed (Figure 3). The tick weight was not affected; the oviposition was reduced by 16% for the polypeptide Bm86 and 14% for the Bm86 antigen compared to the controls. Furthermore, egg fertility was moderately reduced by 11% and 5% for the polypeptide and Bm86, respectively. The overall efficacy of the polypeptide Bm86 was 58%, compared to 57% for the Bm86 antigen, against *R. microplus* infestations.

### 3.3. Humoral Immune Response

**Figure 4:**
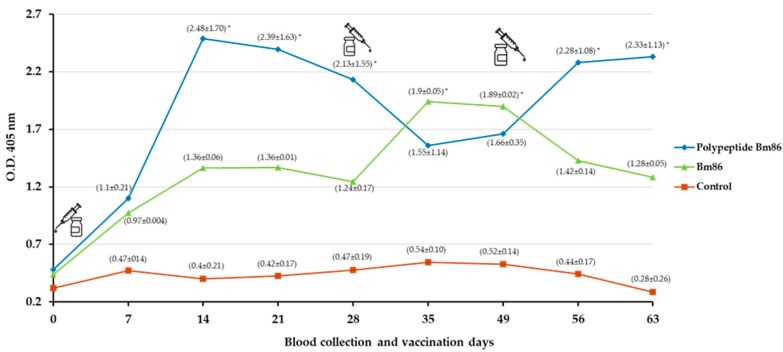
Antibody response in immunized cattle. Bovine serum antibody levels to recombinant antigens were determined by indirect ELISA. Antibody levels in immunized cattle were expressed as the OD_405_ nm value for the 1:100 serum dilution and compared between immunized and control cattle using a one-way ANOVA test (* *p* < 0.05; N = 4). The time of the three immunization shots is indicated.

The humoral immune response of cattle immunized with the polypeptide Bm86 and the formulation containing the Bm86 antigen are shown in Figure 4. The IgG levels in the polypeptide Bm86-immunized animals increased after the first immunization and decreased after the second immunization, achieving at least 2.40 OD units compared to the control group. Cattle immunized with the Bm86 antigen generated a slightly humoral immune response one week after the second booster. However, a significant decline occurred after the third booster obtained, at most, 1.90 OD IgG levels. These results showed that antibody levels were atypical in all animals tested and only were significantly different (*p* < 0.05) on some days of the experiment.

### 3.4. Western Blot Detection of Native Bm86 Protein in Tick Tissues

**Figure 5:**
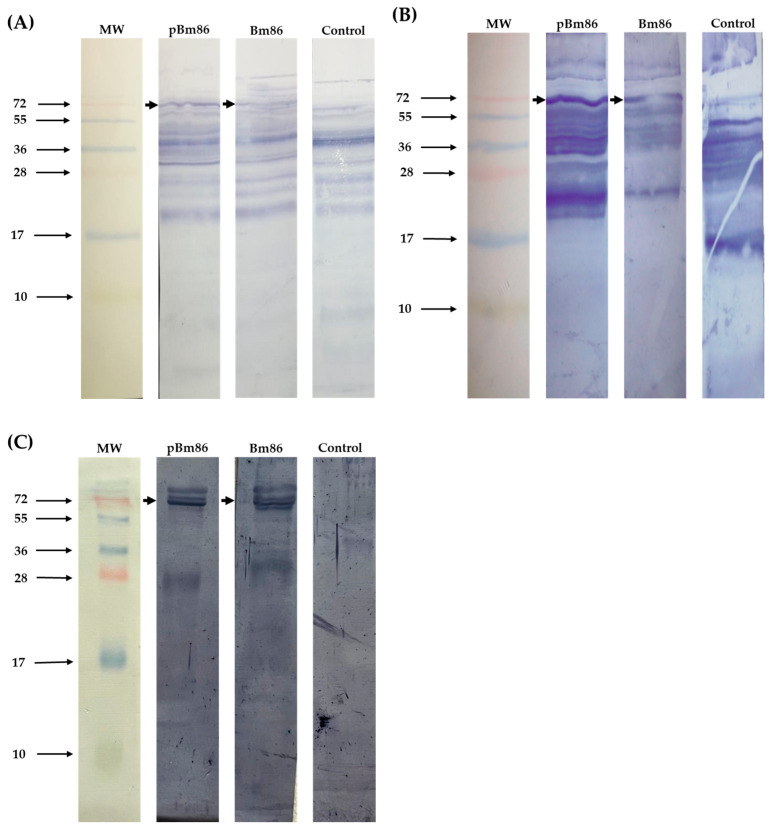
Western blot of *R. microplus* tick tissues probed with anti-Bm86 IgG antibodies. 10 µg of (**A**) egg, (**B**) larvae, and (**C**) guts sections were loaded per well in 15% SDS-PAGE gels and transferred to PVDF membranes. Sera from one representative cow per group were used and developed with an anti-bovine IgG-AP conjugate. Native Bm86 protein is indicated with arrows. MW, molecular weight.

Western blot revealed that sera collected at day 35 from cattle immunized with the 2 antigens recognize the native Bm86 protein compared to the control group (Figure 5). The IgG antibodies produced from one representative cow per group recognized tick proteins derived from engorged *R. microplus* female guts. Furthermore, the antibodies also recognized the native protein in larvae and eggs. The analysis revealed an affinity for a protein of ~72 kDa, similar to the expected molecular weight of the Bm86 protein from the *R. microplus* ticks. In contrast, the native Bm86 protein was not recognized by the serum of the control animal. However, we detected other bands with different molecular weights in tick tissues; possibly, these antibodies were produced before the immunization trial due to previous tick exposure.

### 3.5. Correlation Analysis

**Figure 6:**
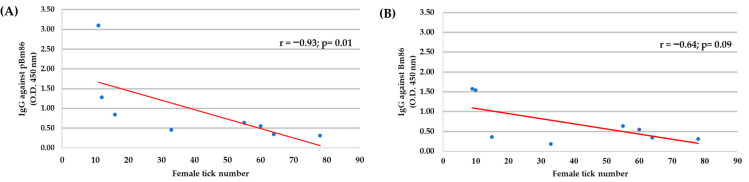
The Spearman’s Rho correlation analyses (*p* < 0.05) were conducted to correlate the IgG antibody levels in immunized cattle with the adult female tick number collected during the experimental trial in individual cattle (N = 8). (**A**) polypeptide Bm86, and (**B**) Bm86. The linear correlation coefficients (r) and *p*-value are shown.

The correlation analysis between the IgG antibody level at day 30, just after the first immunization, and the reduction of tick infestation until the end of the study (day 63) showed a negative correlation for ticks fed on polypeptide Bm86-immunized cattle (r = −0.93, *p* = 0.01), suggesting that the reduction of ticks collected after feeding was the result of the protective antibodies elicited in immunized cattle. However, no correlation was observed in ticks fed on animals immunized with the Bm86 antigen (r = −0.64, *p* = 0.09) (Figure 6).

## 4. Discussion

Developing improved tick vaccines requires the characterization of new candidate tick-protective antigens and the design of more effective and cheaper anti-tick vaccine formulations. Different approaches are currently used in bioinformatics, such as immunoinformatics, based on the application of computational tools to study the molecules of the immune system. These are able to guide the design of experiments to answer important questions in immunobiology and vaccinology [41]. Following this strategy, this study is derived from in silico analysis of antigenic and immunogenic epitopes on the Bm86 protein to develop a conserved recombinant polypeptide using *E. coli* as a heterologous host for protein expression. This system provides an efficient and economically viable method to rapidly produce high quantities of recombinant protein, making it a cost-effective platform for developing immunogens. In contrast, other systems—such as yeast, insect, and mammalian cells— have outstanding protein production capabilities but nevertheless involve expensive development, steep learning curves, and significant standardization costs [42,43,44].

Herein, the polypeptide Bm86 evaluated in cattle against *R. microplus* ticks under field conditions confirmed the immunogenic potential to elicit a humoral immune response. The more affected parameter was the adult female tick number, with a reduction of 44% by the polypeptide Bm86 and 48% by the Bm86 antigen compared to the control group (*p* < 0.05). These findings were within the range reported in previous experiments [19,33,34,45,46]. Interestingly, both experimental groups did not significantly reduce tick weight, oviposition, and egg fertility compared to controls. Although the results achieved by the polypeptide Bm86 were higher in reducing oviposition (16%) and egg fertility (11%), these results contradict other research where reduction of the oviposition has an important effect due to interactions between anti-Bm86 antibodies and *R. microplus* ovary proteins [32,47,48,49]. This finding may be explained by the amount of protective anti-Bm86 antibodies elicited by cattle, sequence divergence, or possibly by recombinant protein folding. Nevertheless, the overall efficacy obtained with the polypeptide Bm86 (58%) and Bm86 antigen (57%) were similar to previous cattle field trials carried out in Argentina, Brazil, Colombia, Cuba, Mexico, and Venezuela, reaching 51–91% efficacy [19,45,46,50].

On the other hand, similar research using synthetic peptides derived from the Bm86 protein reported vaccine efficacy ranging from 70% to 81% [25,32]. In the same way, using P0-Bm86 protein conjugates has demonstrated great immunogenic capacity and obtained 84% vaccine efficacy in a controlled pen trial [48].

Both experimental groups demonstrated an immune response based on concentrations of anti-Bm86 antibody levels. Cattle immunized with the polypeptide Bm86 showed higher anti-pBm86 levels after the first immunization and decreased after the second immunization. However, remained significantly different (*p* < 0.05) compared to the control group, which has been commonly found in previous studies with the Bm86 vaccine [25,32,48,51]. Contrary to the Bm86 antigen results, antibody levels were low in immunized cattle, possibly for different reasons, such as the health condition and the age of the animals used in the experiment [40]. According to Mughini-Gras [52], studies with *Leptospira* spp. vaccines have shown a negative correlation between the age of cows and post-vaccination antibody titers, suggesting that immunosenescence increases in adult cattle over time, resulting in a reduced response to vaccination. Our results agree, since the average age of the cows used was 6.5 years; this probably revealed that older cattle have a weak organism affecting the immune response. It is known that CD4+ cells produce IL-2, a cytokine that induces the differentiation of B cells into antibody-secreting plasma cells. However, with aging, the capacity of CD4+ cells to produce IL-2 gradually decreases, resulting in limited activation of B cells [53]. Consequently, plasma cells are compromised, and the anti-Bm86 antibody production is diminished and shows moderated affinity for the antigen.

Moreover, studies in mice demonstrated that antibodies generated in old mice are less protective than those produced in young mice. In addition, IgG1 plasma cells elicit high-affinity antibodies in young mice, different from aged mice that predominantly produced IgM plasma cells [54]. Another study investigated the relationship between age and leukocyte population during the pre-calving period in cows, finding a lower number of B cells in older cows, which affected the role of antigen-presenting cells, production of antibodies, and the development of memory B cells. Our results are similar to previous research where the number of lymphocytes decreased due to aging, particularly in cows older than 1.5 years. Shihab [55] attributed this to the generation of free radicals that affect the composition of cell membranes and compromise lymphocyte survival.

A negative correlation was found between the number of engorged female ticks and the level of anti-pBm86 IgG antibodies in cattle; this suggests that protective antibodies were directed against tick protein epitopes in the recombinant polypeptide, occasioning a reduced number of adult female ticks collected. These results agree with those reported previously [33,45,48,49] and underline the anti-tick vaccine’s effect on controlling cattle tick infestations. On the other hand, the Bm86-immunized cattle showed low antibody levels and no significant correlation, possibly due to using older cattle in the study.

The Western blot analysis indicates that both antigens are immunogenic and induced protective antibodies; anti-IgG antibodies recognized the native Bm86 protein present in the eggs, larvae, and guts of *R. microplus*, whereas pre-immune sera recognized other native proteins due to the presence of natural antibodies in cattle. Furthermore, the recognition of anti-pBm86 antibodies to larvae and midgut proteins was strongly detected, suggesting a higher expression, similar to those tick antigens previously tested against *R. microplus* tick infestations [39,45,49,56]. In addition, some ticks collected on vaccinated cattle showed marked changes from their standard gray/green color to a characteristic dark-red color, indicating gut damage. The atrophy of guts observed is the same as previous vaccinated cattle studies where ingesting blood containing anti-tick antibodies affected multiple biological processes [40,57,58,59,60] and commonly reduced the biotic potential, resulting in a progressive reduction of tick populations.

Although the conditions present in this study were a field trial in a free-grazing system and with previous exposure to *R. microplus* tick infestations, the results obtained with the polypeptide Bm86 were slightly better than other reports [45,50], probably due to the conserved epitopes considered in the polypeptide design, which are effective to elicit antibody levels and demonstrate immunogenic potential in cattle against *R. microplus* [36]. However, the results reflected the fact that increased age and physiological status of the animals affected the response to the vaccination without forgetting that environmental factors, such as stress and condition, could also be involved in cow-to-cow variation in response to vaccination, as suggested by de la Fuente [61].

Several authors currently follow reverse vaccinology as a better method to produce tick antigens because it offers advantages such as the speed identification and characterization of novel potential tick antigens [28]. Others focus their anti-tick vaccine research on combining antigens (cocktail anti-tick vaccines) or selecting protective peptides that could improve the efficacies of individual vaccines [62,63,64]. Those approaches are important to optimize the efficacy of vaccines, similar to what was done in this study with the polypeptide Bm86 derived from a Mexican *R. microplus* strain. In addition, it is essential to note the need to test immunogens against tick infestations under field conditions because vaccination trials are the main component required to determine if a tick antigen is immunoprotective and effective in reducing tick populations.

Finally, immunological control emerges as a financially feasible and ecologically favorable alternative, with high potential in developing integrated tick management options involving the rational use of acaricides, cultural practices, and/or biological control agents. This all-inclusive strategy aims to reduce the excessive use of acaricides, environmental contamination, and animal/human health risks, following the One Health context [65].

## 5. Conclusions

This study reports the first vaccination trial using a polypeptide Bm86 derived from a Mexican *R. microplus* strain against natural tick infestations. The results allow us to conclude that the polypeptide Bm86 can induce a specific immune response in cattle and confer protection against *R. microplus* in a Mexican tropical region. These findings highlight the importance of considering an integrated management approach for tick control and support further experiments with this antigen to demonstrate its effectiveness as a regional vaccine.

## Figures and Tables

**Table 1:** Efficacy of the polypeptide Bm86 against natural infestations by *Rhipicephalus microplus* ticks.

*Rhipicephalus microplus* (Martínez de la Torre, Veracruz Strain)					
	Percent Reduction (Immunized/Control) ^b^				
Experimental Group ^a^	DT	DW	DO	DF	E ^c^
Polypeptide Bm86	44% * (36 ± 3)	0% (137 ± 44)	16% (60 ± 20)	11% (32 ± 21)	58%
Bm86	48% * (34 ± 2)	0% (175 ± 40)	14% (54 ± 29)	5% (34 ± 23)	57%
Control	(64 ± 10)	(139 ± 41)	(71 ± 29)	(36 ± 17)	-

^a^ Cattle were randomly assigned to experimental groups (N = 4), immunized, and challenged with *R. microplus* (field conditions). ^b^ The percent reduction was calculated concerning the control group: DT, % reduction in tick infestation; DW, % reduction in tick weight (mg); DO, % reduction in oviposition (mg); DF, % reduction in egg fertility (mg). The average ± S.D. for adult female tick number, tick weight, oviposition, and egg fertility are shown in parenthesis and were compared by one-way ANOVA test between immunized and control groups (* *p* < 0.05). ^c^ Vaccine efficacy (E) was calculated as 100 [l−(CRT × CR0 × CRF)], where CRT, CRO, and CRF are the reduction in the number of adult female ticks, oviposition, and egg fertility as compared to the control group, respectively.

## Data Availability

The database and the statistical analyses are available upon reasonable request.
